# Influence of the Applied Protective Coating on the Technological Parameters of the Moulding or Core Sand Surface

**DOI:** 10.3390/ma17235737

**Published:** 2024-11-23

**Authors:** Mariusz Łucarz, Dariusz Drożyński, Karolina Kaczmarska, Alena Pribulová, Peter Futáš

**Affiliations:** 1 Faculty of Foundry Engineering, AGH University of Krakow, Reymonta 23 St., 30-059 Krakow, Poland; dd@agh.edu.pl (D.D.); krlnk@agh.edu.pl (K.K.); 2 Faculty of Materials, Metallurgy and Recycling, Technical University of Kosice, Letna 1/9, 042 00 Kosice, Slovakia; alena.pribulova@tuke.sk (A.P.); peter.futas@tuke.sk (P.F.)

**Keywords:** protective coating, moulding sand, casting defects, surface quality, adhesion, viscosity, permeability, abrasiveness

## Abstract

An important issue in obtaining a good casting surface without defects in contact with the liquid metal is the use of protective coatings on the surface of the moulding sand forming the mould cavity. Protective coatings are based on highly refractory materials that are finely ground and protect the moulding sand from thermal stresses from the molten metal. An important aspect of obtaining the appropriate properties of the protective coatings used is the method of application. This article presents the results of a quality study of the protective coatings obtained, which were applied by dip and painting with a brush. Four different coatings were applied to mould sand samples prepared on grain matrices with different average grain sizes. During the tests, the viscosity of the protective coatings, their gas-forming capacity, sieve analyses of the grain matrix used, the permeability of the moulding sand with the protective coating and the abrasiveness and adhesion of the protective coatings were determined. The quality of the coating obtained was found to depend on its type, the size of the grain matrix used in the moulding sand and the method of application. The experiments carried out indicate that a comparative study of the available protective coatings can indicate the best solution, taking into account the grain matrix used and the method of application adopted.

## 1. Introduction

The application of protective coatings is a common procedure to increase the resistance of the surface layer of objects to dynamic forces, weather conditions, or temperature. Their role is to increase resistance to abrasion, corrosion phenomena and the effects of high temperatures. The protective coating is also intended to form a barrier against complex interactions on the surface, e.g., friction (consequently abrasion) and the accompanying heat generation, or counteract oxidation as the temperature rises. In some cases, a protective coating may be applied to selected areas of the surface that are particularly exposed to excessive external influences. There are also a number of methods and ways to apply protective coatings to the surface to be protected.

The most recognisable protective coating procedure is the prevention of corrosion on steel surfaces. In this case, two types of galvanisation are encountered: the application of varnish coatings or both together. Zinc coatings can be formed on the metal surface by galvanising or hot-dip galvanising. The issues of creating appropriate protective coatings during hot-dip galvanising, including the method of creating the appropriate galvanising bath, its modification and specific working conditions, are discussed by the authors in previous works [1,2,3,4,5,6,7,8,9,10,11,12]. The method of applying coatings by current and current-less galvanising is presented in other works [13,14,15]. Another treatment to protect against corrosion is the application of protective varnish coatings. The formation of various lacquer protective coatings operating in different corrosive environments has been characterised in previous work [16,17,18,19,20,21,22,23]. Paint protective coatings do not only prevent corrosion. Special solutions for high-temperature protection are being developed, as presented in previous work [24]. Another area of application for paint coating is the creation of a radiation phenomenon to deflect heat and provide protection against the heating of the roof surfaces [25].

Protective coatings applied to metal surfaces are also a wear protection measure. The most commonly used methods are surfacing, spray, plasma and laser. These are used to apply various compositions of elements to harden the surface layer of a component that is subjected to wear processes (adhesive, erosive and abrasive) [26,27,28,29,30,31,32,33,34].

In the foundry industry, protective coatings are used to protect the surface of moulds from excessive exposure to the high temperature of the casting alloy. They consist of a refractory matrix, a high-temperature bonding material, a suitable solvent and other additives. The fluidity to apply them smoothly to the surface of the core or mould can be achieved with water. Then, such a surface must be dried. In the case of organic solvents, the coating can be additionally dried by firing. Protective coatings used in the foundry industry are multicomponent materials and are produced in solid, paste and liquid consistencies. The protective coating on the casting mould fulfils a number of tasks. Its role is to reduce the roughness of the casting surface. An important function is also to prevent the moulding materials from burning into the casting. The protective coating also makes it easier to remove the finished casting during the mould removal process. The protective effect in the mould is achieved by covering the pores of the moulding sand, which prevents liquid metal from penetrating deep into the moulding or core sand. The use of a protective coating also prevents the formation of folds and cracks. A good protective coating should have the following characteristics [35]:

- The refractoriness of the coating must be higher than the temperature of the liquid metal.
- Adequate strength properties.
- Be characterised by resistance to cracking during drying or curing.
- The coating must not be reactive with metal oxides at elevated temperatures.
- Should have the ability to resist erosion of the mould-filling metal.

The main components forming the protective coating matrix of the foundry are graphite, coke, quartz SiO_2_, zircon ZrSiO_2_, sintered magnesite MgO, talc 3MgO-4SiO_2_-H_2_O, chamotte 3Al_2_O_3_-SiO_2_, alumina Al_2_O_3_ and muscovite KAl_2_(OH,F)_2_|(AlSi_3_O_10_). In addition to water (protective water-based coating), the following are used to dilute protective coatings: ethyl alcohol C_2_H_5_OH, isopropyl alcohol CH_3_CHOHCH_3_, methyl alcohol CH_3_OH, butyl alcohol CH_3_CH_2_CH_2_CH_2_OH and methylene chloride ZrSiO_2_ [35].

Protective coatings for sand and metal moulds and cores are applied by dipping, brushing, pouring and spraying. The type of application used depends on whether the coating is waterless or water-based. The distribution of the protective coating is determined by the size of the casting mould component and the production volume. For small cores, application by dip and brush painting is suggested, and for medium and large cores, all of the above-mentioned methods are suggested, with pouring and spraying more commonly used for high-volume production due to lead times [35].

In 2023, the global metal casting market reached a value of approximately $162.68 billion, with an anticipated annual growth rate of about 3.8% through 2032. A significant portion of these castings, especially within sectors such as automotive, construction and machinery, is produced using sand moulds, which represent the most popular casting method. In 2023, the sand-casting segment alone was valued at around $83.72 billion, underscoring its versatility and cost-effectiveness, particularly for large and complex components [36,37]. Sand-casting technology is one of the most widely used casting processes in the world, especially in the production of large and complex components. It is estimated that sand castings account for about 60–70% of global casting production [35,36,37]. Thermal expansion of foundry sand is the main factor that influences casting defects. The foundry industry uses quartz sand for economic reasons. It is a highly refractory material, which is an advantage, but it also has a significant disadvantage from the perspective of making a good mould. It is characterised by discontinuous thermal expansion at elevated temperatures, accompanied by a large change in volume of up to 3.9%. This phenomenon occurs during the thermal phase transformation of α-quartz into β-quartz at a temperature of approximately 573 °C [35,38,39,40,41]. If quartz sand is contaminated with compounds from binding materials (K, Na from water glass and alkaline resin components), the volume may change by a further 15% in contact with high temperatures [35,38,42,43]. In addition to volumetric changes in the grain itself, the changes may relate to the dilatation between the various mould components (core and casting mould), the grain composition of the sand, its shape, the type and amount of binder, the moulding sand additives used and the degree of compaction. Studies on the use of different sands and their propensity to form casting defects are presented in [44,45,46], where most of the theory is based on heat stress in silica sands.

Protective coatings for casting moulds play a key role in the casting production process, influencing their quality and durability [47,48,49]. A previous article [49] discusses various casting coating technologies, highlighting their importance in minimising casting defects and protecting moulds from high temperatures and aggressive chemicals. These coatings are designed to provide an effective protective barrier to increase the efficiency of the casting process. References [50,51] highlight the ecological benefits of using aqueous coatings, which enhance casting quality while also being environmentally sustainable.

The variety of applications of protective coatings can also be seen in studies on casting defects, such as inclusions in sand and metal penetration [48]. These coatings can be based on different materials, allowing their properties to be adapted to the specific requirements of the casting process. Ongoing research demonstrates the importance of water-based coatings in the context of iron castings, which require special protection [51,52]. Furthermore, studies on moisture migration in the surface layers of sand moulds [53] and the influence of gaseous atmospheres on coating properties [54] highlight the importance of process parameters in achieving optimal results.

The subject of water- and alcohol-based protective coatings was characterised in previous papers [55,56,57,58,59,60]. Research mainly concerns the drying kinetics and the selection of a suitable protective coating in contact with a given liquid casting alloy. An important aspect of the protective coating is the selection of a suitable composition [61]. Another area of research concerns the protective coating that separates the liquid-casting alloy from the metal mould [62].

The main objective of this research was to find out what properties the various protective coatings supplied for the moulding of sand have. The analyses concerned the coatings themselves, as well as the technological aspect after application to the surface of the moulding sand. As the tests were carried out on reference moulds, which can be defined as small cores, two application methods were used: dipping and brush painting. Furthermore, the quality of the protective coatings was analysed by applying them to moulding sand of the same composition, but with different grain sizes. In this case, we checked how the obtained intergrain space influences the technological parameters of the prepared moulding sand-protective coating compositions in the studied range.

## 2. Materials and Methods

### 2.1. Protective Coating

Four different protective coatings were tested, the characteristics of which are shown in Table 1.

We used a light green protective coating based on sintered magnesite and alcohol as a carrier liquid. Due to its long flow effect, it is particularly suitable for pouring coated cores.

First, tests were performed on the protective coatings themselves: viscosity, gas formability and thermogravimetric analysis. The last two tests were carried out on the cured coatings obtained by drying them by burning them.

The determination of the viscosity of protective coatings was carried out using a modified RHEOTEST^®^ RV2 (RHEOTEST, Ottendorf-Okrilla Germany) apparatus. The modification consisted of the use of computer control of the apparatus and, at the same time, the recording of measurement data. We used a rotational rheometer with coaxial cylinders, with the outer one (stationary) serving as the measuring vessel and the inner one (movable) connected via a shaft to a cylindrical coil spring allowing the determination of the torsional moment induced by the viscosity of the liquid under test. During the viscosity determination, the S/S1 measuring cylinder system was used. The dynamic viscosity value *η* is read off the graph as the tangent of the angle formed by a line drawn to decrease the rotational speed of the rotameter. The value is read from the linear function for the determined shear stress *τ* for the given shear rates *γ˙*. Extrapolating the line formed to intersect the tangential stress axis *τ* determines the flow limit *τ_B_*. The tangent of the angle of inclination formed by the extrapolated line with the shear rate axis *γ˙* determines the viscosity *η*. The corresponding relationship is shown by Equation (1), in which *γ˙_x_* is the shear rate, *τ_B_* is the extrapolated yield stress (shear stress) and *τ_x_* is the shear stress occurring at the shear rate *γ˙_x_.*

(1) $$\eta =\frac{\tau_{x}-\tau_{B}}{\dot{y_{x}}}$$

**Figure 1:**
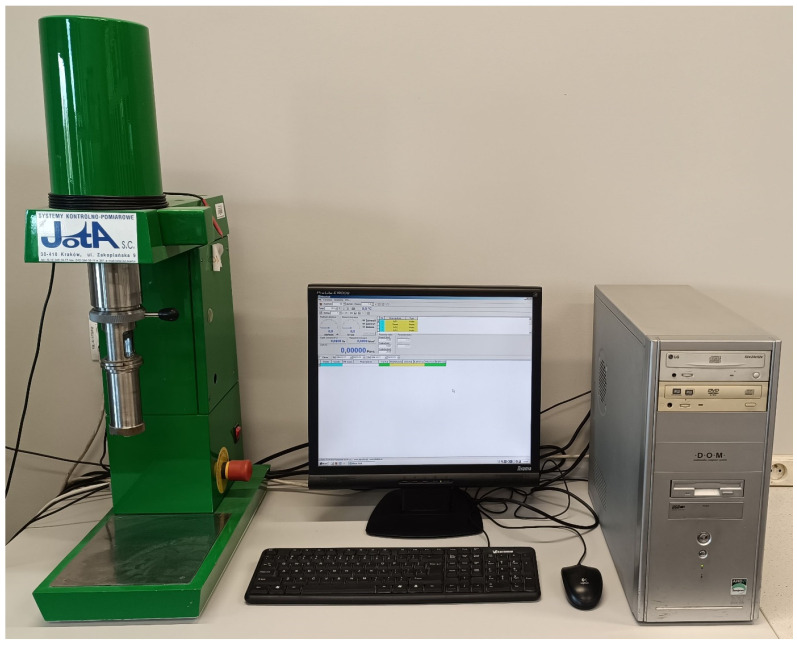
Viscosity test rig.

The viscosity test stand is shown in Figure 1.

**Figure 2:**
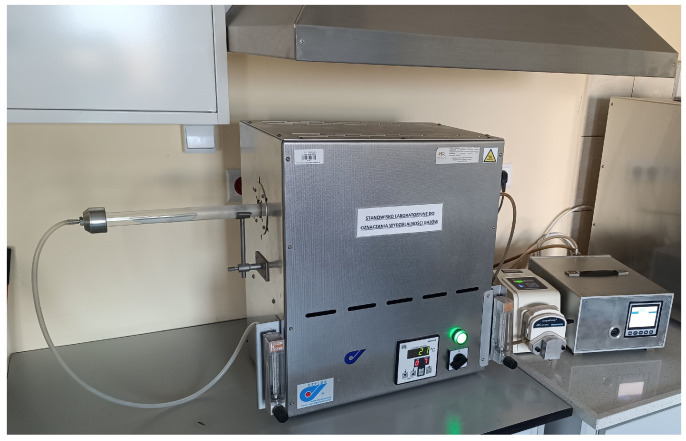
Gas-generating testing station.

Gas-activity measurements of material samples were carried out on the test stand shown in Figure 2, equipped with a tubular furnace type PRC 30M/1300 from CZYLOK, Jastrzębie—Zdrój, Poland, a peristaltic pump type BT100-2J from LongerPump, Hebei, China, and a control and recording unit.

The measurement consisted of heating a tube furnace (quartz tube) to a temperature of 1000 °C, into which a ceramic boat with a sample of the material under investigation weighing approximately 2 g (weighed to an accuracy of ±0.001 g) was then introduced (outside the heating zone). After the sample of material to be analysed had been introduced, the tube was sealed on one side and connected to a peristaltic pump on the other, to create negative pressure in the reactor and pump out the resulting gases. The sample was then introduced into the heating zone, where it was heated very quickly to the measurement temperature and the amount of gas formed was recorded. The gas-forming results are presented as the averages of three determinations. The results of the measurements were converted to one gramme of sample moulding sand.

In order to assess the protective coatings more fully, a thermal analysis test was performed. The oven was heated at a rate of 10 °C/min to a temperature of 1000 °C, at which point the organic compounds should have been fully degraded and destroyed. The test was carried out using a TA Instruments SDT Q600 thermal analyser (DSC/TGA), New Castle, DE, USA. The mass of the samples subjected to TG thermal analysis was approximately 2 g. Aluminium oxide crucibles were used for the measurements, which allowed measurements up to 1500 °C.

### 2.2. Moulding Sand with Protective Coatings

The first test was a sieve analysis of the individual grain matrices. The analysis was carried out according to PN-83/H-11077 [63] on a set of sieves with a mesh clearance of 1.6, 0.8, 0.63, 0.40, 0.32, 0.20, 0.16, 0.10, 0.071 and 0.056 mm (and bottom). The test was carried out on two parallel samples of 50 g. A laboratory shaker LPzE-2e from Multiserw-Morek, Marcyporęba, Poland was used. The sieving time was 900 s.

To determine the permeability of the protective coatings, cylindrical mouldings were first prepared from a furfuryl resin-bonded moulding sand. To evaluate the behaviour of protective coatings applied to the surface of the fittings, two moulding sands, M1 and M2, were made using grain matrices that differ in grain size. Coarse quartz sand S1 (SIBELCO, Bukowno, Poland) was used for the first moulding sand M1, and fine quartz sand S2 (Grudzeń-Las, Sławno, Poland) was used for the second moulding sand M2. The composition of the prepared moulding sand is shown in Table 2.

The matrices used were subjected to sieve analysis, the results of which are shown in Table 3.

**Figure 3:**
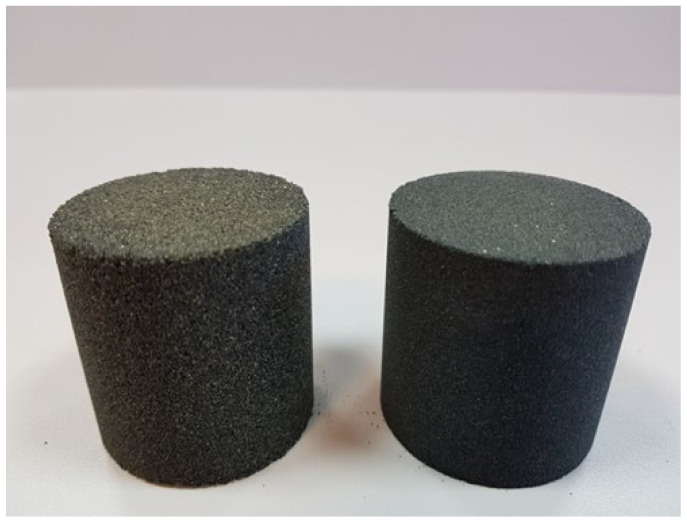
Test pieces: coarse sand S1 (left) and fine sand S2 (right).

Both moulding sand types used for making cylindrical shapes were prepared in a laboratory rotary mixer type LM-1 produced by the Experimental Department of the Foundry Institute in Krakow. First, a weighed portion of the matrix was poured into the mixer, to which the hardener was dosed and stirred for 90 s, then the resin was dosed and stirred for another 90 s. The moulding sand thus prepared was poured into moulds reproducing cylindrical shapes (diameter ϕ = 50 mm, height h = 50 mm) for the determination of permeability *P^u^*, abrasion A and adhesion *N_f_*. The moulds were mounted on an apparatus for vibratory compaction of moulding sand, type LUZ-1 made by WADAP Wadowice, which was turned on for a period of 15 s using a maximum vibration amplitude of 2 mm. After compaction, the extension was removed, the excess moulding sand was cut off and the main part of the moulding sand was taken apart. The prepared shapes were placed aside to cure under ambient conditions, which were as follows: temperature *T_e_* ≈ 16 °C, relative humidity *W_w_* ≈ 50%. Figure 3 shows the finished cylindrical shapes for the two grain matrices used.

In the study, two methods of applying protective coatings were used, namely, brush application and dipping. Applying four different coatings using the two methods to mould parts made of different matrices resulted in 16 combinations, making it necessary to introduce a designation for each option.

**Figure 4:**
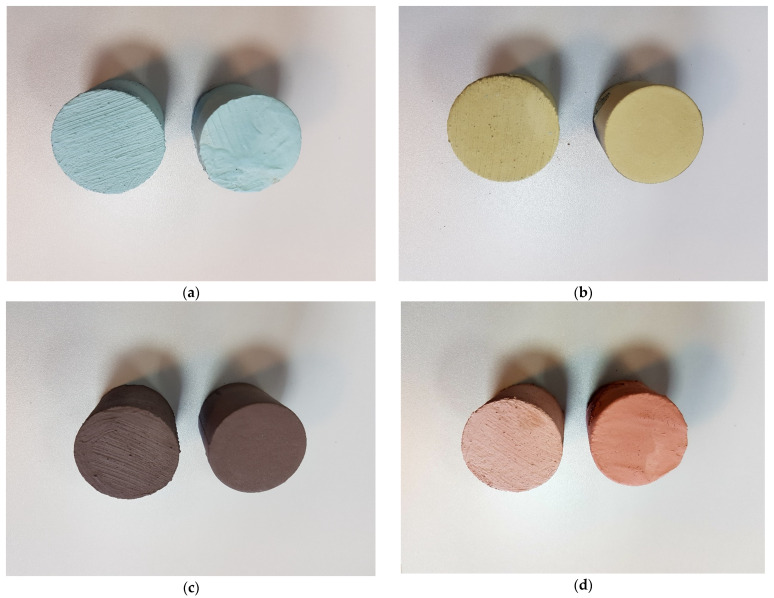
Samples after coating application: (**a**) P1; (**b**) P2; (**c**) P3; (**d**) P4; left sample was painted with a brush; right sample was casting dipped.

A four-character symbol was proposed, in which the first two characters, e.g., P1, refer to the type of coating (as described in Table 1), the third character indicates the method of coating application (D—dipping, B—brushing) and the fourth and fifth characters indicate the type of moulding sand (M1 moulding sand on matrix S1—coarse sand, M2 moulding sand on matrix S2—fine sand). Figure 4 shows the test specimens prepared accordingly.

The permeability of *P^u^* in the dried state was determined using the rush method on an LPiR-1-type apparatus manufactured by WADAP Wadowice. The protective coatings were applied to the base of the cylindrical shapes after curing. After the protective coating was applied to the surface of the fitting, it was dried by burning off the solvent. Permeability was measured 24 h after application of the coating. The cylindrical fitting was mounted on a special sleeve with an inflatable rubber gasket inside, which was pressed against the side of the fitting with compressed air using a hand pump. The permeability for each protective coating and application method was determined on three fittings, and the results are the arithmetic mean of these measurements.

**Figure 5:**
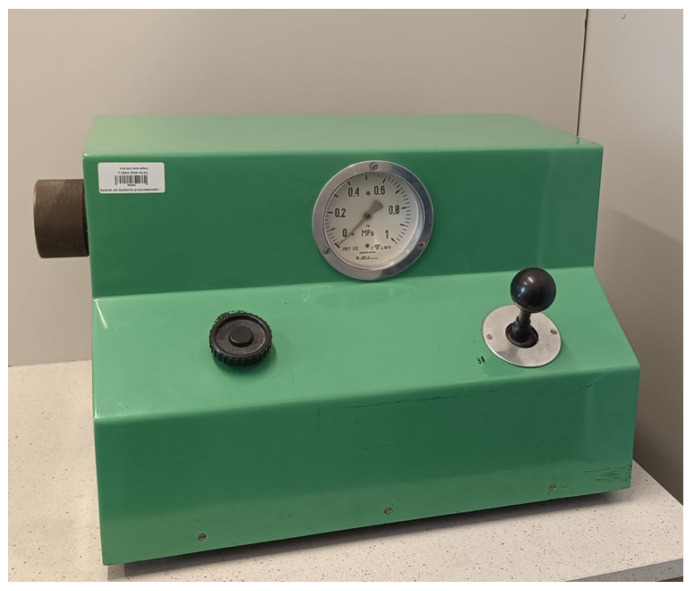
Test stand for adhesion of protective coatings.

The determination of the adhesion of protective coatings *N_p_* was carried out on an apparatus type LPP produced by WADAP Wadowice according to the standard BN-80/4024-04 [64]. The principle of the test is to determine the pressure at which the coating applied on a standardised cylindrical moulding breaks when subjected to blowing with compressed air. The surface of the coating after curing should be free from cracks and scratches. The fittings prepared in this way are placed in the sleeve insert of the adhesion test apparatus so that the surface of the fitting with the coating is on the side of the protective mesh of the apparatus. The insert with the moulded part is placed in the sleeve of the apparatus. The approximate air pressure at which the coating fails is determined first in one of the fittings. During the test, the fitting is blown with compressed air at 0.05 MPa and the pressure is then increased in steps of 0.025 MPa until the coating fails. On the other five fittings, the test starts with a compressed air pressure of 0.025 MPa lower than that at which the coating failure occurred on the test fitting. The value of the failure pressure readout is a measure of the *N_p_* adhesion of the applied protective coating. The adhesion test apparatus is shown in Figure 5.

The abrasiveness was determined on fittings prepared in the same way as for the permeability determination. The only difference was that in the case of the abrasiveness test, the coating was applied to the side of the cylindrical mould.

**Figure 6:**
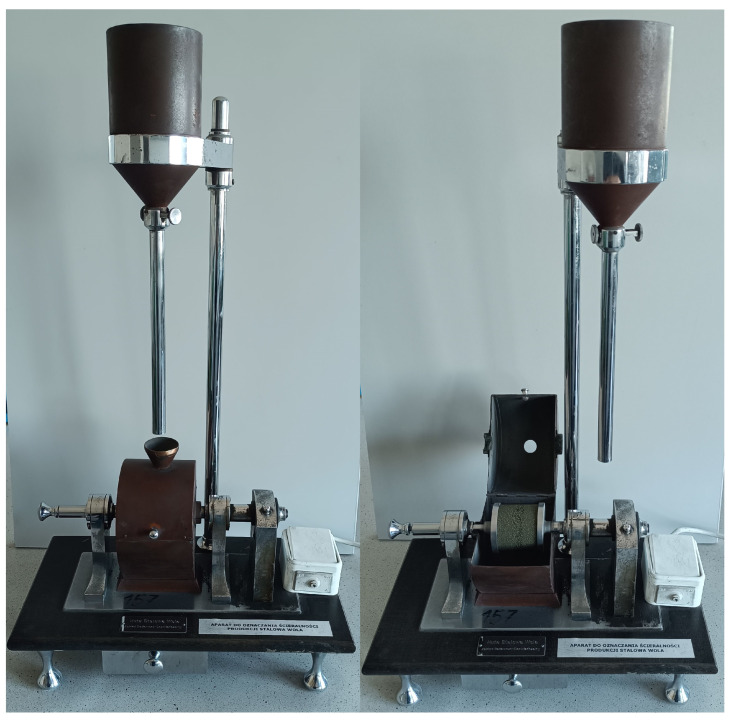
Test stand for abrasion testing of protective coatings *A_HSW_*.

The abrasiveness *A_HSW_* of the tested moulding sands was determined according to the BN-77/4024-02 standard [65]. Measurements were taken on an apparatus manufactured by Huta Stalowa Wola (HSW). The measurement consisted of mounting a weighed cylindrical mould (cured for 24 h) coated with the coating under test on the apparatus holder. The shaper was then made to rotate at 1 rpm by an electric motor. During the determination, a shot falls onto the rotating shaper from a height of 307 mm and causes abrasion. A 1750 g steel shot, 1 mm in diameter, weighed to the nearest 1.0 g, was used. The test rig is shown in Figure 6.

The abrasiveness of the *A_HSW_* was determined as the difference between the initial sample mass *m_f_* and the post-test sample mass *m_e_* by the initial value *m_f_* according to Equation (2)

(2) $$A_{HSW}=\frac{m_{f}-m_{e}}{m_{f}}\cdot 100$$

**Figure 7:**
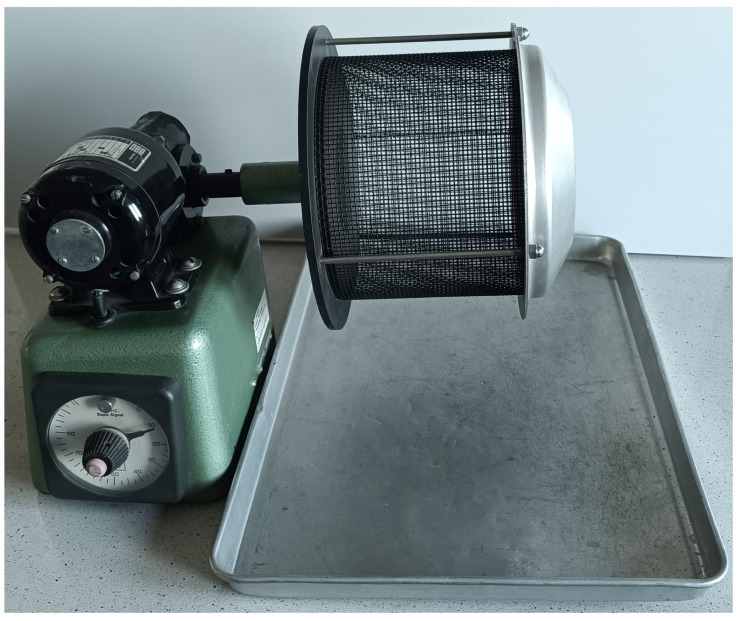
*A_Wf_* abrasion test bench for protective coatings.

Since, in the above method, the action of the abrasive agent is limited to a narrow area of the moulded part, a second method for assessing the abrasiveness of coatings was proposed using the *A_Wf_* moulding index apparatus. The apparatus is shown in Figure 7. Measurement using this method involves placing a cylindrical moulded part with a protective coating of *m_f_* weighed to the nearest 0.01 g in the drum sieve of the apparatus. The sieve is inclined at an angle of 7° to the horizontal, has a diameter of 178 mm, a mesh clearance of 2.4 mm and rotates at 57 rpm during the measurement. The entire abrasion measurement (rubbing of the shaper in the sieve) lasted 60 s. After the rubbing was completed, the shaper was weighed *m_e_* and the percentage weight loss was calculated (3).

(3) $$A_{Wf}=\frac{m_{f}-m_{e}}{m_{f}}\cdot 100$$

Measurements with both methods were carried out on three fittings, and the results presented are the arithmetic mean of these measurements.

**Figure 8:**
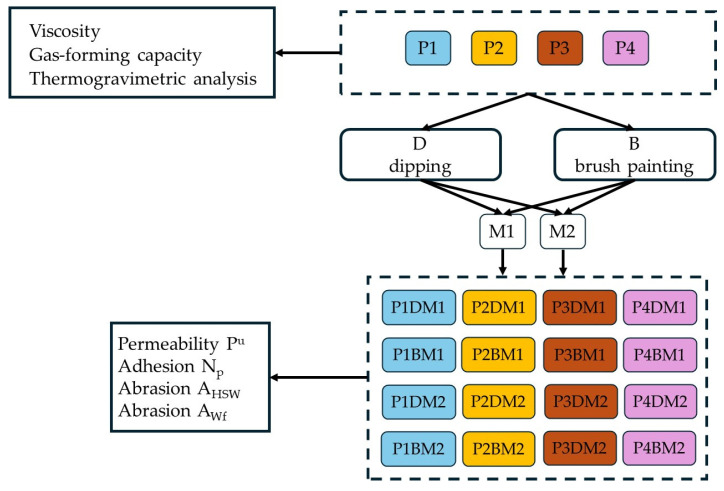
Flowchart of the research programme.

The programme of completed studies is shown in Figure 8.

## 3. Results

**Figure 9:**
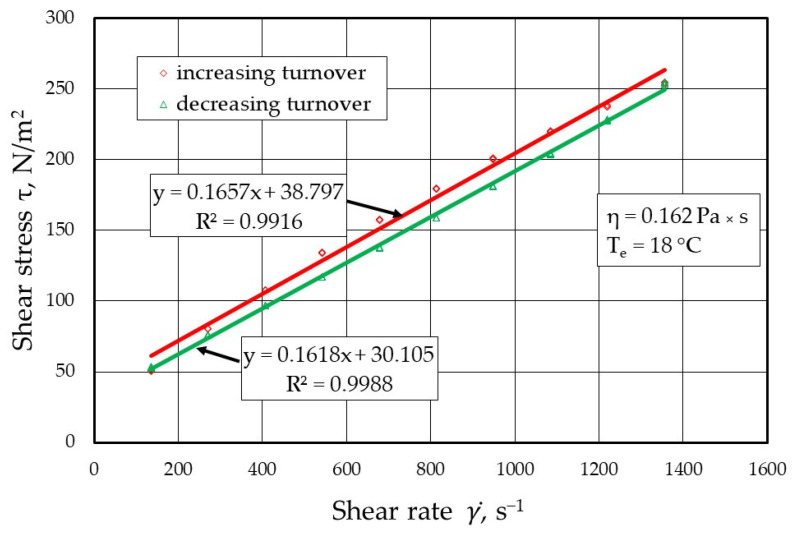
Melt flow curves $\tau =f(\dot{\gamma )}$ for protective coating P1 (ISOMOL 0267).

**Figure 10:**
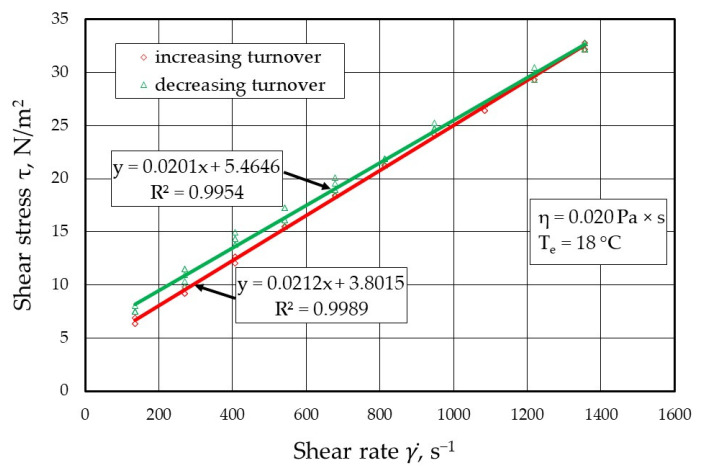
Melt flow curves $\tau =f(\dot{\gamma )}$ for protective coating P2 (MAGNESITSCHLICHTE 5848).

**Figure 11:**
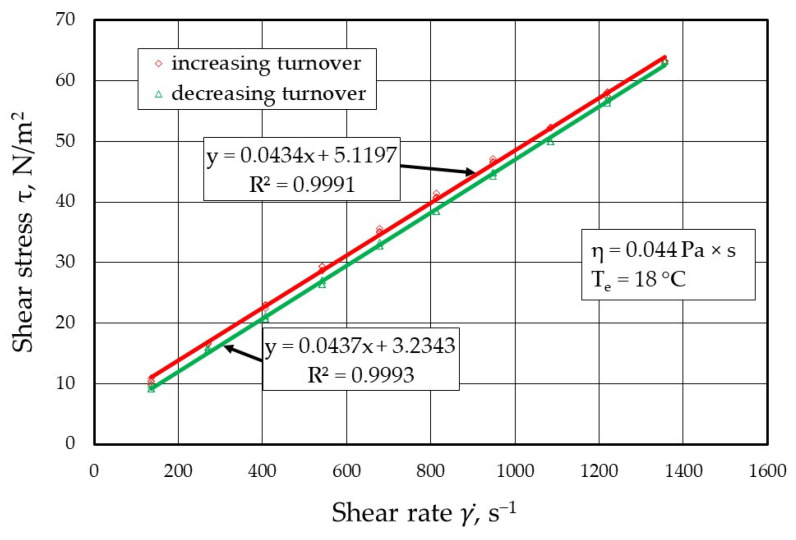
Melt flow curves $\tau =f(\dot{\gamma )}$ for protective coating P3 (TENOTEC 7804A).

**Figure 12:**
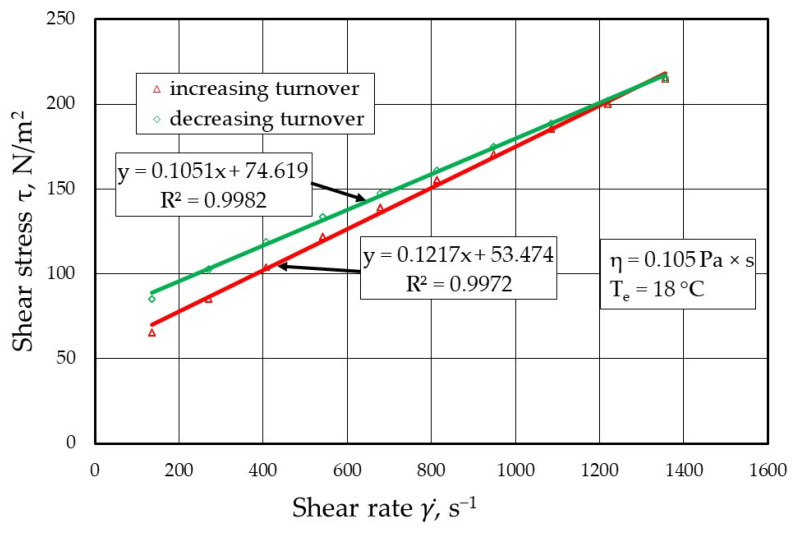
Melt flow curves $\tau =f(\dot{\gamma )}$ for protective coating P4 (TENOCOATING).

**Figure 13:**
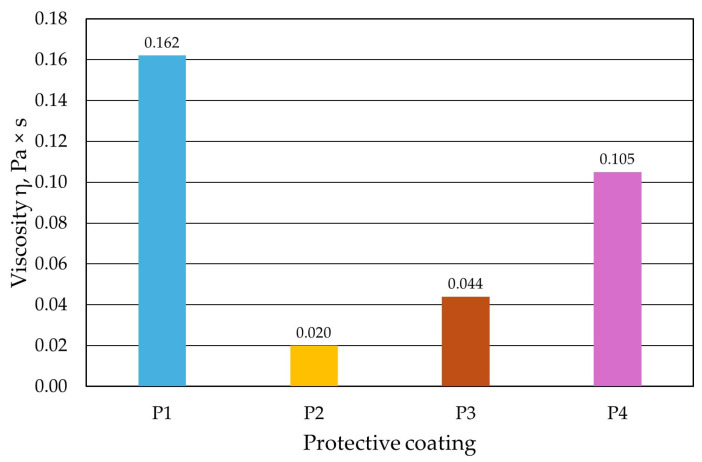
Summary of dynamic viscosity *η* of tested protective coatings.

Figure 9, Figure 10, Figure 11 and Figure 12 show the results of the dynamic viscosity measurements of the protective coatings as delivered by the manufacturer on the device shown in Figure 1. The results obtained from the dynamic viscosity *η* of the tested protective coatings determined at an ambient temperature *T_e_* of approximately 18 °C are summarised in Figure 13.

As can be seen, each protective coating had a different dynamic viscosity *η*. It is assumed that the higher the viscosity, the denser the fluid, and consequently, the protective coating is less fluid. In view of this, the tested coatings can be characterised as the coating labelled P2 as being the most fluid and the protective coating P1 being the least. The dynamic viscosity *η* of the protective coatings influences their application on the tested mouldings and, as a result, can influence the formation of a specific coating quality on the moulding surface.

**Figure 14:**
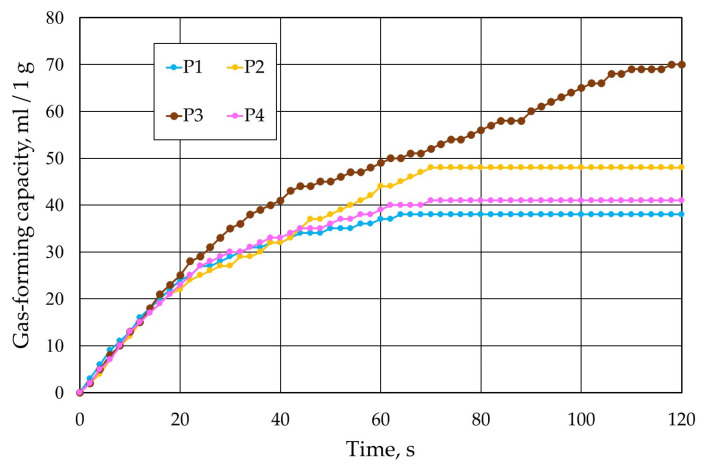
Summary of results of gas formability of tested protective coatings.

Important parameters for moulding materials are their gas formability and resistance to high temperatures. Therefore, protective coatings were characterised in this respect. The solvent in the coatings was alcohol, so their drying was performed by burning off the diluent. After drying, the solid samples were subjected to a gas-forming test. The results of the tests performed are shown in Figure 14.

**Figure 15:**
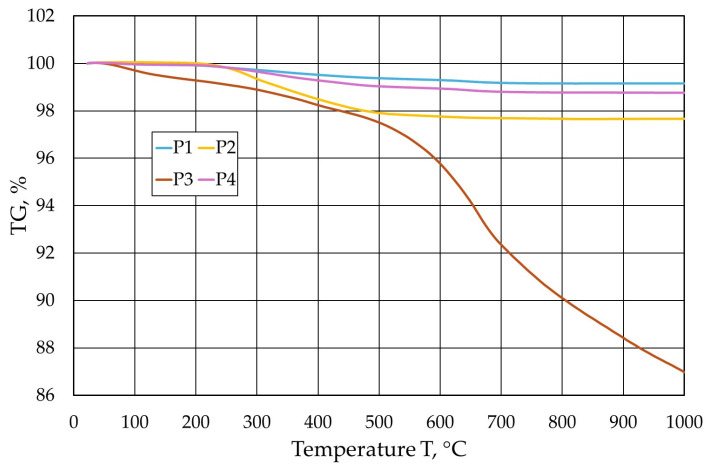
Summary of results of thermogravimetric TG analysis of tested protective coatings.

Based on the analyses performed, it was found that the least gas-forming volatile parts at a temperature of 1000 °C were generated by the P1 protective coating and the most by the P3 protective coating. The gas-forming results are reflected in the thermogravimetric analysis performed. Figure 15 shows the results of the realised analyses of the individual protective coatings.

The results of the thermogravimetric analysis obtained allow us to conclude that the least amount of organic matter is in the P1 coating, as a slight loss in its mass was registered. On the other hand, the most organic components were found in the P3 coating.

The casting mould is subjected to high temperatures after being flooded with liquid metal. Since the binder that binds the grain matrix is of organic origin, the moulding sand formed from the combination of these two components undergoes a gradual process of degradation (gasification) and destruction (burning) in contact with the liquid alloy. Each organic binder has its own thermostability, which depends on its composition. Therefore, in order to increase the thermal resistance of the moulding sand against the dynamically influencing high-temperature casting alloy, protective coatings made of refractory materials are applied to the casting mould. The task of protective coatings in the first instance is to reduce the destructive effect of the high-temperature moving metal.

**Figure 16:**
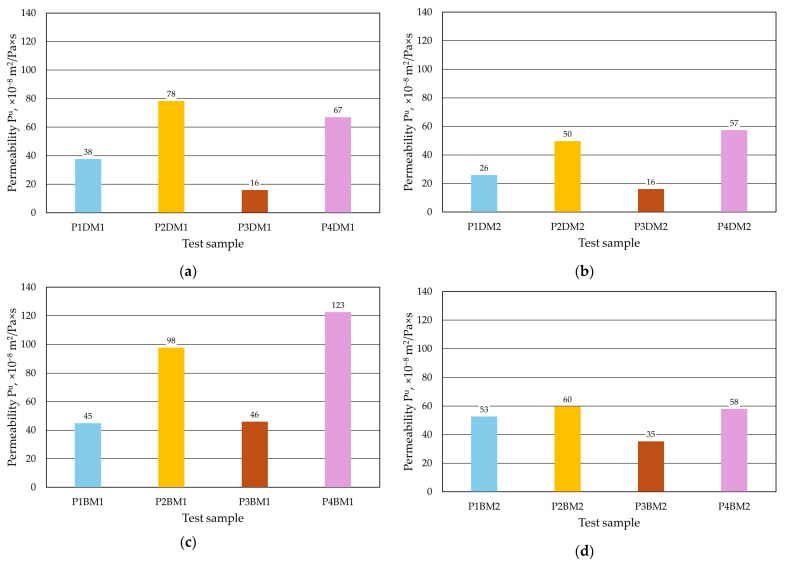
Permeability *P^u^* of moulding sand with protective coating: (**a**) M1 moulding sand with dip coating, (**b**) M2 moulding sand with dip coating, (**c**) M1 moulding sand with brush coating and (**d**) M2 moulding sand with brush coating.

In order to select the best coating, tests were carried out by applying protective coatings to the cylindrical samples in different ways. An important parameter for the quality of the prepared mould, and therefore the moulding sand, is its *P^u^* permeability. Tests were carried out on moulding sands that differed in grain size. The larger the grain size of the matrix, the larger the intergrain spaces, which improves the mould permeability and migration of the resulting gases at the metal–mould interface. A coarser grain matrix is used for larger castings, where the amount of mass is greater, and therefore greater mass gas formation is expected. Hence, there is a need for larger intergrain spaces for gas evacuation. This study was carried out to determine whether the application of a protective coating on moulding sand of different grain sizes while maintaining the same moulding sand composition has a significant effect on its permeability with a given coating. The results of the experiments are shown in Table 4 (dipping), Table 5 (brushing) and Figure 16.

As expected, lower permeability was recorded for protective coatings applied to mouldings with a finer grain matrix in M2 moulding sand. The results obtained were consistent for both coating application methods used, whether by immersion (Figure 16a,b) or by brush (Figure 16c,d). In contrast, the individual protective coatings had different effects on the moulding sand parameter studied. The P2 and P4 coatings had the highest permeability, while the P3 protective coating had the lowest. It can also be seen that the application method has a significant effect on permeability. It was found that applying the coating by immersion reduces the permeability of moulding sand with a protective coating (cf. Figure 16a with Figure 16c and Figure 16b with Figure 16d). This is due to a more gedcomplete filling of the intergrain spaces of the top layer of the moulding with the protective coating and a more uniform distribution of the coating layer on its surface.

**Figure 17:**
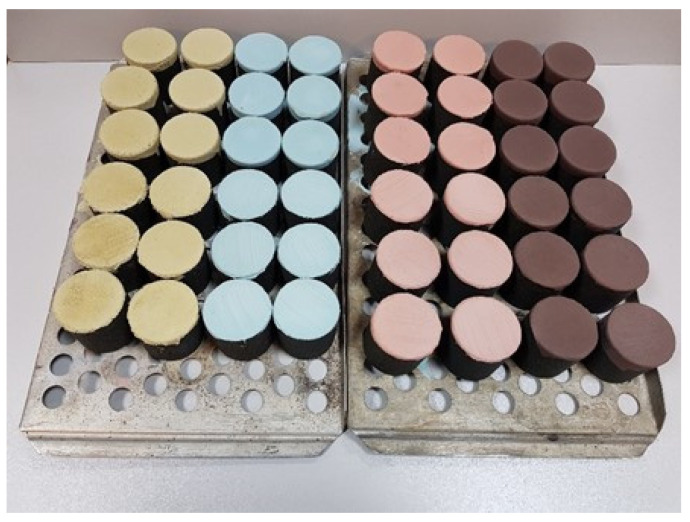
Samples prepared for adhesion testing of *N_p_* protective coatings.

**Figure 18:**
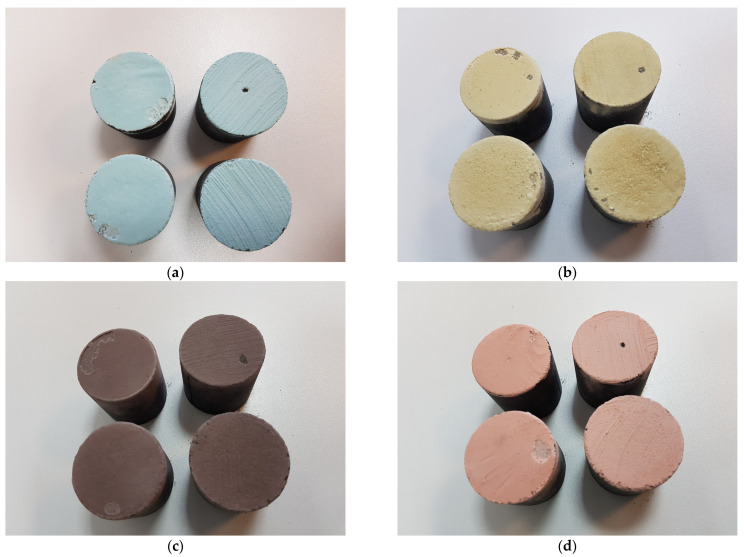
Samples with protective coatings after adhesion test *N_p_*: (**a**) protective coating P1, (**b**) protective coating P2, (**c**) protective coating P3, (**d**) protective coating P4; left side of coating applied by dipping, right side of coating applied by brush painting.

**Figure 19:**
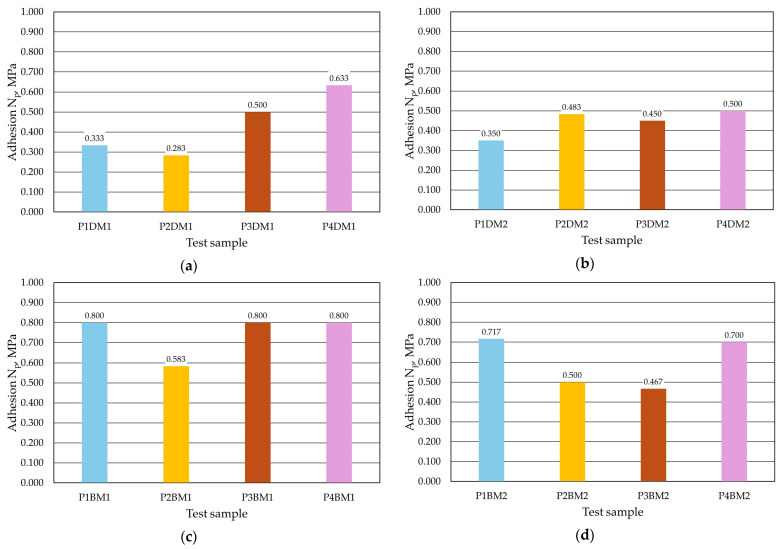
Adhesion *N_p_* of the protective coating to the moulding sand: (**a**) the dip-coated M1 moulding compound, (**b**) the dip-coated M2 moulding compound, (**c**) the brush-coated M1 moulding compound and (**d**) the brush-coated M2 moulding compound.

The next test was to check the adhesion of the *N_p_* coating to the moulding sand samples. Figure 17 shows the samples prior to the test. The better quality of the protective coating obtained by dipping is noticeable. In contrast, Figure 18 shows how the samples look after the test. The images show peeling of the protective coating, which results in a loss of integrity of the applied layer. On individual protective coatings, the loss of cohesion occurs at different locations on the surface of the test specimen. The resulting *N_p_* adhesion measurements are shown in Table 6 (dipping), Table 7 (brushing) and Figure 19.

For this study, it was found that the protective coating adhered better to the moulding sand if applied by brush painting. Higher adhesion values were obtained regardless of the size of the grain matrix used in the moulding sand. When the protective coating was applied by brushing, higher adhesion *N_p_* was obtained for the sand moulding with the larger grain matrix M1.

**Figure 20:**
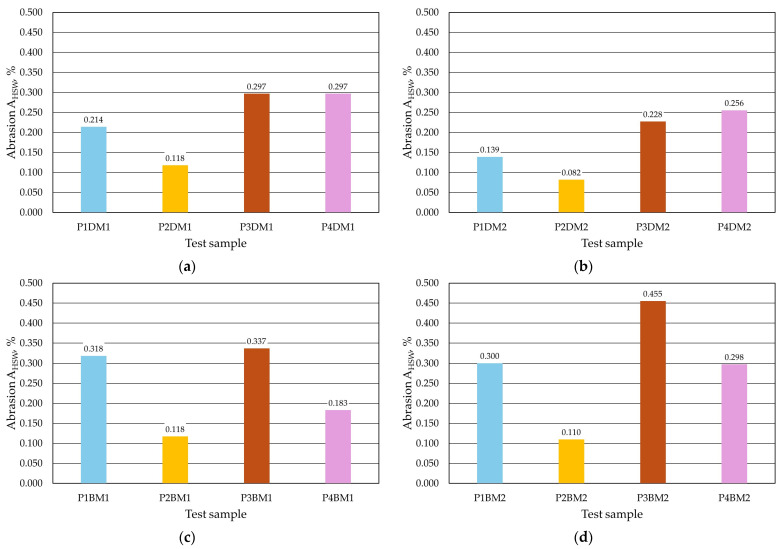
Abrasiveness *A_HSW_* of the protective coating: (**a**) M1 moulding sand with dip coating, (**b**) M2 moulding sand with dip coating, (**c**) M1 moulding sand with brush coating and (**d**) M2 moulding sand with brush coating.

**Figure 21:**
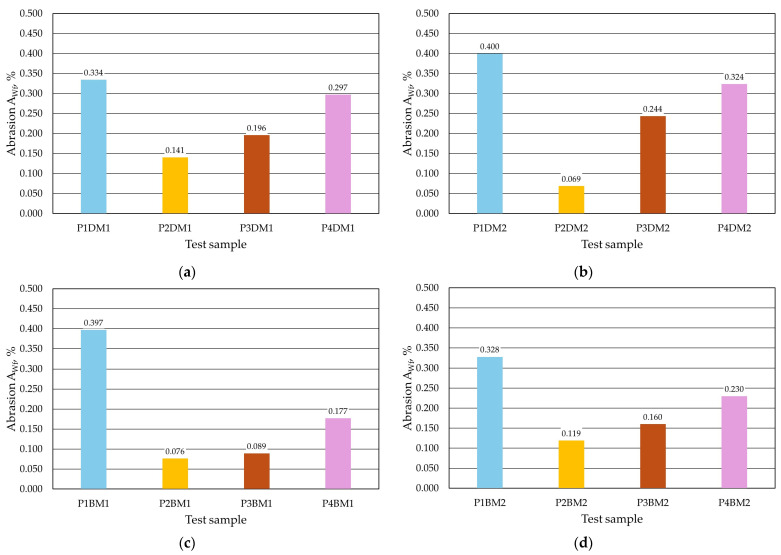
Abrasiveness *A_Wf_* of the protective coating: (**a**) M1 moulding sand with dip coating, (**b**) M2 moulding sand with dip coating, (**c**) M1 moulding sand with brush coating and (**d**) M2 moulding sand with brush coating.

The quality of the protective coatings obtained was also assessed by testing their abrasion resistance. This parameter was tested using two methods. The results for the first method, denoted by the parameter *A_HSW_*, are shown in Table 8 (dipping), Table 9 (brushing) and Figure 20, and the second method, denoted as *A_Wf_*, is shown in Table 10 (dipping), Table 11 (brushing) and Figure 21.

The *A_HSW_* abrasion test, which involves local abrasion of the protective coating with a steel shot, indicates that the method of application of the protective coating has an impact on the parameter under study. Lower sample weight losses were obtained for protective coatings applied by immersion. Protective coating P2 has the highest abrasion resistance and coating P3 the lowest. The influence of matrix size is also noticeable. Smaller percentage weight losses were found for the finer matrix used in the M2 moulding sand when the coating was carried out by immersion. For brush application of the coating, no clear effect of the sand matrix grain size on _AHSW_ abrasion was obtained.

The second abrasion test, which involved abrading the protective coating from the cylindrical surface of the sample, showed that abrasion depends on the dynamic viscosity *η*. The greater the viscosity and, at the same time, the density of the protective coating tested, the greater the sample loss (cf. Figure 13 and Figure 21), i.e., the poorer the quality of the protective coating surface obtained. In this test, the highest abrasiveness was found for the protective coating and the lowest for protective coating P2. The *A_Wf_* abrasion test indicates the advantage of the protective coating application method by brush painting.

When analysing many protective covers, it is difficult to clearly indicate unambiguously which is the best solution. For this reason, a point classification was introduced for each parameter analysed, assuming four points for the best value and one point for the worst value. The sum of the points forms a ranking of the quality of the protective coatings. The parameters studied were divided into three groups: the first is about the tests of the protective coatings themselves, the second concerns the quality of the protective coatings on the M1 mould sand and the third is regarding the protective coatings on M2 moulding sand.

In the first of the criterion groups tested, it was assumed that the lower the dynamic viscosity, the better the quality of the coating. For the gas-formability, the best coating was assumed to be the one with the lowest gas emission. On the other hand, for thermogravimetric analysis, the coating that had the lowest percentage weight loss of the test sample was considered the best. The corresponding classification is shown in Table 12.

The tests carried out on the protective coatings indicated that the protective coating proved to be the best solution in this criterion group and was assigned the highest number of points.

The subsequent criterion groups were based on the same scores. *P^u^* permeability was best when the value obtained was the highest. Also, the highest value for adhesion *N_p_* was the expected value. For the *A_HSW_* and *A_Wf_*, the smallest value indicated a better coating. Table 13 summarises the results for moulding sand M1, which used a larger grain matrix. Table 14, on the other hand, is for moulding the M2 moulding sand with a finer grain matrix.

Combined in one group, the technological properties indicated that the P2 coating would be the best for the application, scoring the highest values in the overwhelming number of parameters tested. The P1 coating was rated the worst in this case.

This enhanced performance of the M2 moulding sand with the finer grain matrix aligns with findings on how nanomechanical properties are influenced by the internal structure of sand matrices. Magazzù et al. [66] demonstrated that nanomechanical characteristics, such as elasticity and stiffness, are highly dependent on the particle arrangement within the matrix, with finer structures yielding improved mechanical stability and performance. Xu et al. [67] further support this by showing that complex, fine-grained structures in sand moulds contribute to better material responses under stress, enhancing the uniformity and durability of coatings applied. These insights suggest that the superior results observed with the P2 protective coating on M2 sand are likely due to the nanomechanical advantages provided by the finer grain matrix, which facilitates the better adhesion, permeability and abrasion resistance of the coating. By comparing all the results obtained, it is possible to indicate that protective coating P2 is the best one to use.

## 4. Conclusions

The presented tests of a selected group of different protective coatings lead to several conclusions. It should be noted that the results presented concern the coatings in the state supplied by the manufacturer, i.e., without any interference with their condition.

It was found on the basis of the tests that the lower the viscosity of the protective coating, the higher the permeability of the moulding sand because the density is lower, which creates the possibility of gas migration through the given moulding sand. The P3 coating showed a deviation from this rule, which confirms the need to verify protective coatings before they are used in the foundry. Gas-forming tests performed on the protective coatings after the coatings were dried by firing and thermogravimetric analysis indicated that this coating contained significant amounts of organic components, relative to the other materials compared. This condition may have formed a deviation from the perceived rule for the other coatings.

The adhesion of protective coatings is dependent on the size of the grain matrix and the method of application. Larger matrix grains in the moulding compound increase adhesion. As far as the application method is concerned, there is a noticeable advantage to painting with a brush. This was confirmed for all protective coatings used.

The abrasiveness of protective coatings, i.e., the resistance to dynamic impact, is lower for coatings with lower dynamic viscosity. This was confirmed by a test carried out on samples that were abraded over the entire surface. At the same time, the application method shows an advantage in abrasion resistance when the sample is painted with a brush. The application of the protective coating with a certain pressure exerted by the brush increases the resistance to abrasion. At the same time, this does not affect the permeability of the moulding compound. In the case of point dynamic impact on a protective coating-moulded sand sample, the abrasion results obtained were inconclusive.

In casting production, surface quality also plays an important role. In this case, the method of application of dipping the moulded part in the protective coating showed an advantage.

Future research should focus on testing protective coatings under real industrial conditions, with attention to their impact on casting surface quality and durability under prolonged exposure to molten metal. Additionally, exploring the ecological aspects of commercially used coatings could guide the selection of materials with a lower environmental impact while maintaining high effectiveness in mould protection.

## Figures and Tables

**Table 1:** Characteristics of the protective coatings used in the study.

Label	Name	Characteristics
P1	ISOMOL 0267 (Foseco, Vesuvius Moravia Sro, Trinec, Czech Republic)	A high-quality alcohol-based coating specifically designed for the flow coating of moulds and cores. Suitable for use on moulds and cores bonded using silicates or organic resins. Although specifically designed for steel casting production of any section thickness, it is equally effective in other ferrous and non-ferrous applications.
P2	MAGNESITSCHLICHTE 5848 (Hüttenes-Albertus GmbH, Düsseldorf, Germany)	It is a light green protective coating based on sintered magnesite and alcohol as a carrier liquid. Due to its long flow effect, it is particularly suitable for cores coated by pouring.
P3	TENOTEC 7804A (Foseco, Vesuvius Moravia Sro, Czech Republic)	A high-viscosity universal coating, based on magnesium-iron silicate and modified with carbon and iron oxide. Is suitable for moulds and cores in all conventional moulding materials. The range of applications includes moulds and cores of any size in grey, nodular and malleable iron as well as heavy and light metals.
P4	TENOCOATING (Foseco, Vesuvius Moravia Sro, Czech Republic)	A soft, smooth paste for moulds and cores with a typical odour of alcohol. Is a pasty dispersion of refractory filler materials in an organic solvent. The solvent is volatile and highly inflammable. The range of applications includes moulds and cores for the lightest to the heaviest castings of steel as well as grey, nodular and malleable cast irons as well as heavy and light metals.

**Table 2:** Composition of moulding sand for protective coatings.

Sand Label	Sand Matrix S1 (Coarse Quartz) wt.%	Sand Matrix S2 (Fine Quartz) wt.%	Resin Ratio wt.%	Hardener Ratio wt.%
M1	100	-	1.2	0.3
M2	-	100	1.2	0.3

**Table 3:** Parameters of the grain matrix applied to the moulding sand.

Sand Label	Sieve Analysis Parameter	Arithmetic Mean of Grain *d_a_* mm	Average Grain Size *D*_50_ mm	Major Fraction *F_g_* %
S1	0.40/0.32/0.20	0.40	0.39	91.82
S2	0.20/0.10/0.16	0.22	0.21	88.98

**Table 4:** Permeability P^u^ of moulding sand with protective coating (dipping).

Statistic	Permeability *P^u^* ×10^—8^ m^2^/Pa × s							
	P1DM1	P2DM1	P3DM1	P4DM1	P1DM2	P2DM2	P3DM2	P4DM2
Max	0.366	0.182	0.448	0.376	0.175	0.120	0.252	0.349
Min	0.115	0.055	0.194	0.234	0.000	0.040	0.201	0.146
Average	0.214	0.118	0.297	0.285	0.139	0.082	0.228	0.256
Standard Deviation σ	0.134	0.064	0.134	0.079	0.036	0.041	0.026	0.103

**Table 5:** Permeability P^u^ of moulding sand with protective coating (brushing).

Statistic	Permeability *P^u^* ×10^—8^ m^2^/Pa × s							
	P1BM1	P2BM1	P3BM1	P4BM1	P1BM2	P2BM2	P3BM2	P4BM2
Max	0.367	0.228	0.417	0.276	0.371	0.147	0.535	0.363
Min	0.270	0.055	0.232	0.062	0.201	0.054	0.400	0.263
Average	0.318	0.118	0.337	0.183	0.300	0.110	0.455	0.298
Standard Deviation σ	0.049	0.096	0.095	0.110	0.089	0.049	0.070	0.057

**Table 6:** Adhesion *N_p_* of the protective coating to the moulding sand (dipping).

Statistic	Adhesion *N_p_* MPa							
	P1DM1	P2DM1	P3DM1	P4DM1	P1DM2	P2DM2	P3DM2	P4DM2
Max	0.350	0.300	0.500	0.700	0.400	0.500	0.500	0.800
Min	0.300	0.250	0.500	0.600	0.300	0.450	0.400	0.500
Average	0.333	0.283	0.500	0.633	0.350	0.483	0.450	0.600
Standard Deviation σ	0.029	0.029	0.000	0.058	0.050	0.029	0.050	0.173

**Table 7:** Adhesion *N_p_* of the protective coating to the moulding sand (brushing).

Statistic	Adhesion *N_p_* MPa							
	P1BM1	P2BM1	P3BM1	P4BM1	P1BM2	P2BM2	P3BM2	P4BM2
Max	0.800	0.650	0.800	0.800	0.800	0.550	0.500	0.750
Min	0.800	0.500	0.800	0.800	0.650	0.450	0.400	0.600
Average	0.800	0.583	0.800	0.800	0.717	0.500	0.467	0.700
Standard Deviation σ	0.000	0.076	0.000	0.000	0.076	0.050	0.058	0.087

**Table 8:** Abrasiveness *A_HSW_* of the protective coating (dipping).

Statistic	Abrasiveness *A_HSW_* %							
	P1DM1	P2DM1	P3DM1	P4DM1	P1DM2	P2DM2	P3DM2	P4DM2
Max	0.366	0.182	0.448	0.376	0.175	0.120	0.252	0.349
Min	0.115	0.055	0.194	0.234	0.000	0.040	0.201	0.146
Average	0.214	0.118	0.297	0.285	0.139	0.082	0.228	0.256
Standard Deviation σ	0.134	0.064	0.134	0.079	0.036	0.041	0.026	0.103

**Table 9:** Abrasiveness *A_HSW_* of the protective coating (brushing).

Statistic	Abrasiveness *A_HSW_* %							
	P1BM1	P2BM1	P3BM1	P4BM1	P1BM2	P2BM2	P3BM2	P4BM2
Max	0.367	0.228	0.417	0.276	0.371	0.147	0.535	0.363
Min	0.270	0.055	0.232	0.062	0.201	0.054	0.400	0.263
Average	0.318	0.118	0.337	0.183	0.300	0.110	0.455	0.298
Standard Deviation σ	0.049	0.096	0.095	0.110	0.089	0.049	0.070	0.057

**Table 10:** Abrasiveness *A_Wf_* of the protective coating (dipping).

Statistic	Abrasiveness *A_Wf_* %							
	P1DM1	P2DM1	P3DM1	P4DM1	P1DM2	P2DM2	P3DM2	P4DM2
Max	0.366	0.182	0.448	0.376	0.175	0.120	0.252	0.349
Min	0.115	0.055	0.194	0.234	0.000	0.040	0.201	0.146
Average	0.214	0.118	0.297	0.285	0.139	0.082	0.228	0.256
Standard Deviation σ	0.134	0.064	0.134	0.079	0.036	0.041	0.026	0.103

**Table 11:** Abrasiveness *A_Wf_* of the protective coating (brushing).

Statistic	Abrasiveness *A_Wf_* %							
	P1BM1	P2BM1	P3BM1	P4BM1	P1BM2	P2BM2	P3BM2	P4BM2
Max	0.367	0.228	0.417	0.276	0.371	0.147	0.535	0.363
Min	0.270	0.055	0.232	0.062	0.201	0.054	0.400	0.263
Average	0.318	0.118	0.337	0.183	0.300	0.110	0.455	0.298
Standard Deviation σ	0.049	0.096	0.095	0.110	0.089	0.049	0.070	0.057

**Table 12:** Classification of the properties of protective coatings.

Label	η Pa × s	Gas-Forming mL/1 g	TG %	Result
P1	1	4	4	9
P2	4	2	2	8
P3	3	1	1	5
P4	2	3	3	8

**Table 13:** Classification of technological properties of protective coatings applied to M1 moulding sand prepared on a larger grain matrix.

Label	*P^u^* 10^−8^ m^2^/Pa × s		*N_p_* MPa		*A_HSW_* %		*A_Wf_* %		Result
	D	B	D	B	D	B	D	B	
P1	2	1	2	4	3	2	1	1	16
P2	4	3	1	3	4	4	4	4	27
P3	1	2	3	4	2	1	3	3	19
P4	3	4	4	4	2	3	2	2	24

**Table 14:** Classification of technological properties of protective coatings applied to M2 moulding sand prepared on a smaller grain matrix.

Label	*P^u^* 10^−8^ m^2^/Pa × s		*N_p_* MPa		*A_HSW_* %		*A_Wf_* %		Result
	D	B	D	B	D	B	D	B	
P1	2	2	1	4	3	2	1	1	16
P2	3	4	3	2	4	4	4	4	28
P3	1	1	2	1	2	1	3	3	14
P4	4	3	4	3	1	3	2	2	22

## Data Availability

The original contributions presented in this study are included in the article. Further inquiries can be directed to the corresponding author.
